# Describing the persistence landscape for introducing microbes into complex communities

**DOI:** 10.1128/aem.00930-26

**Published:** 2026-07-22

**Authors:** Jason E. McDermott, William C. Nelson, Amy E. Zimmerman, Winston Anthony, Devin Coleman-Derr, Joshua R. Elmore, Tara Nitka, Ryan S. McClure, Pubudu P. Handakumbura, Adam M. Guss, Travis J. Wheeler, Robert G. Egbert

**Affiliations:** 1 Biological Sciences Division, Pacific Northwest National Laboratory 536904, Richland, Washington, USA; 2 Department of Molecular Microbiology and Immunology, Oregon Health and Science University 547642, Portland, Oregon, USA; 3 Plant Gene Expression Center, USDA-ARS, Albany, California, USA; 4 Plant and Microbial Biology Department, University of California 1438, Berkeley, California, USA; 5 Environmental Molecular Sciences Division, Pacific Northwest National Laboratory 6865, Richland, Washington, USA; 6 Biosciences Division, Oak Ridge National Laboratory 551173, Oak Ridge, Tennessee, USA; 7 College of Pharmacy, University of Arizona 8041, Tucson, Arizona, USA; Michigan State University, East Lansing, Michigan, USA

**Keywords:** fitness landscape, engineered organism, community, persistence, microbiome

## Abstract

Introducing non-native organisms into natural or designed microbial communities holds enormous potential but faces challenges in establishment, persistence, and containment. We propose the “persistence landscape”—the functional composition of a target microbiome that promotes or discourages engineered organism persistence. Building on existing ecological concepts, this framework predicts the fitness of introduced organisms. Application of this concept would enable controlled introduction of desired taxa and phenotypes, addressing pressing challenges in agronomy, biomanufacturing, and human health.

## COMMENTARY

Naturally occurring microbes have demonstrated great potential in laboratory studies to promote functions beneficial to host ecosystems, such as the human gut or plant rhizosphere (1, 2). Enhancing native phenotypes or incorporating new functions by genome engineering for agronomic, biomanufacturing, or medical applications holds promise to provide ecosystem services that have been lost or are in high demand (2, 3), or to enable robust production of desired molecules (4, 5). However, the performance of introduced microbes in complex environments has been variable due to the difficulties and uncertainties around achieving stable, long-term persistence of the introduced species in the target community (6). For example, genome engineering to control persistence may also reduce overall fitness and diminish or eliminate function in natural environments. Environmental persistence is known to be influenced by multiple factors, including biotic interactions such as competition, predation, and resource exchange, and evolutionary forces such as mutation or horizontal gene transfer (1), as well as abiotic conditions (e.g., pH, temperature, and nutrient availability). How these factors influence each other and the introduced organism, as well as how they function to promote or inhibit its persistence in natural or designed community environments, is poorly understood, especially at the molecular level. While ecological theories exist that address aspects of this topic, there is not a unified framework for understanding the persistence of introduced organisms.

In this perspective, we consider the most important factors that impact the persistence of introduced microbial functions (engineered or otherwise) in a natural or designed environment and the control and containment of introduced organisms within that target environment. We describe a persistence landscape, which extends the concept of a functional community landscape to explicitly represent the persistence of an introduced organism in a target community.

## PERSISTENCE OF ENGINEERED ORGANISMS

The increasing pace of major breakthroughs in engineering microbes and proteins presents unprecedented opportunities to deploy microbes with beneficial functions in the environment (3) or as components of designed communities with specific biomanufacturing or other applications (7). For example, advances in protein and pathway engineering enabled by DNA-editing technologies (2, 8) have enabled scientists to implement these engineering solutions in synthetic biology workhorses like *Escherichia coli* or *Pseudomonas putida* and non-model environmental isolates (9–12). However, an intellectual framework is needed to guide the technical and ethical application of synthetic biology to microbial community engineering.

A number of studies have highlighted the challenges and complexities associated with predicting the persistence of engineered organisms in natural environments. On one hand, incorporation of a genetically tagged *Pseudomonas fluorescens* SBW25 into a soil microbiome showed that the cells persisted up to 250 days after sowing (13). However, another study showed that bioengineered bacteria introduced into an aquatic microcosm decreased below detection limits after 32 days (14). Similar variability in many other studies of bacterial persistence in natural environments demonstrates our lack of understanding important questions about what drives environmental persistence for introduced microbial functions (1). Designed communities have been shown to be capable of increased production rates and robustness for biomanufacturing processes (4, 5, 7, 15). However, the principles that lead to stable incorporation of an engineered organism in such a consortium remain largely unknown.

These studies highlight the challenges in understanding the persistence of engineered microbes and the functions they are designed to carry out in natural or designed environments. Biotic factors are highlighted as a gap in the understanding of organism establishment, with metabolic exchange and antagonistic interactions being prioritized as critical opposing functional factors to be considered (15, 16). They also highlight the need for theory and models that enable targeted strategies—such as ecological matching or functional complementarity—to improve the stability and integration of engineered bacteria in complex environments (17, 18).

## MODELING PERSISTENCE IN A REAL ENVIRONMENT

Community ecological theory, largely developed to understand establishment and dynamics of communities of macroscopic eukaryotic organisms, fails to account for very important aspects of microorganisms such as the rapid rates of reproduction and evolution, phage predation, and molecular interactions between organisms including metabolic exchange and horizontal gene transfer (17, 18). Ecological niche theory defines the conditions, resources, and constraints that allow organisms to survive and reproduce in a system, but microbes do not always follow the assumptions of niche theory, and time scales relevant to microbial generations may mean that the relevant principles may not have time to be realized (17).

Specifically considering the molecular level, the fitness landscape concept was first proposed in the 1930s as a way to describe how genetic differences in a single organism result in novel phenotypes that impact the organism’s fitness under defined environmental conditions (19). In the classic genetic fitness landscape metaphor, the landscape represents the high-dimensional space of possible genotypes, where similar genotypes are positioned closer together and the height of the surface reflects the organism’s fitness, defined as the relative success of its phenotype under a given set of conditions. This conceptual framework provides powerful insights into evolutionary processes, such as adaptive trajectories and constraints (20), and can reveal genetic interactions, such as epistasis, where combinations of genes affect phenotype and fitness in non-additive ways (21). However, the fitness landscape is primarily concerned with the fitness of an individual organism and does not consider the complex interactions that are possible between organisms in a community.

Community function landscapes (22) extend the idea of the fitness landscape to microbial ecosystems to understand a collective function. In the community function landscape, species composition and abundance form a landscape where similar combinations of organisms are close together and disparate combinations are far apart, while functional output (e.g., carbon source utilization) forms the height of the landscape, analogous to fitness in a classic fitness landscape described above. Thus, the landscape describes the emergent functional outcomes of a microbial community such as the ability to metabolize specific substrates determined by the community’s composition and genetic potential (23). Importantly, recent work in this area has revealed that the principle of global epistasis allows determination of linear equations that accurately represent how a single species affects the emergent functional outcome (21, 22). These functional effect equations provide a tractable way of determining a community function landscape based on a limited number of data points.

Community function landscapes reveal how species interactions (e.g., cross-feeding and competition) and environmental conditions govern functional capacities and sensitivities of microbial communities, providing valuable insights into ecological assembly and biotechnological interventions. For instance, studies applying the functional landscape framework to engineered communities have identified critical species, metabolic dependencies, and interaction sensitivities that drive specific outcomes under different environmental conditions (22, 23). However, community function landscapes have not been applied to the understanding of engineered organism persistence.

## THE PERSISTENCE LANDSCAPE

**Fig 1:**
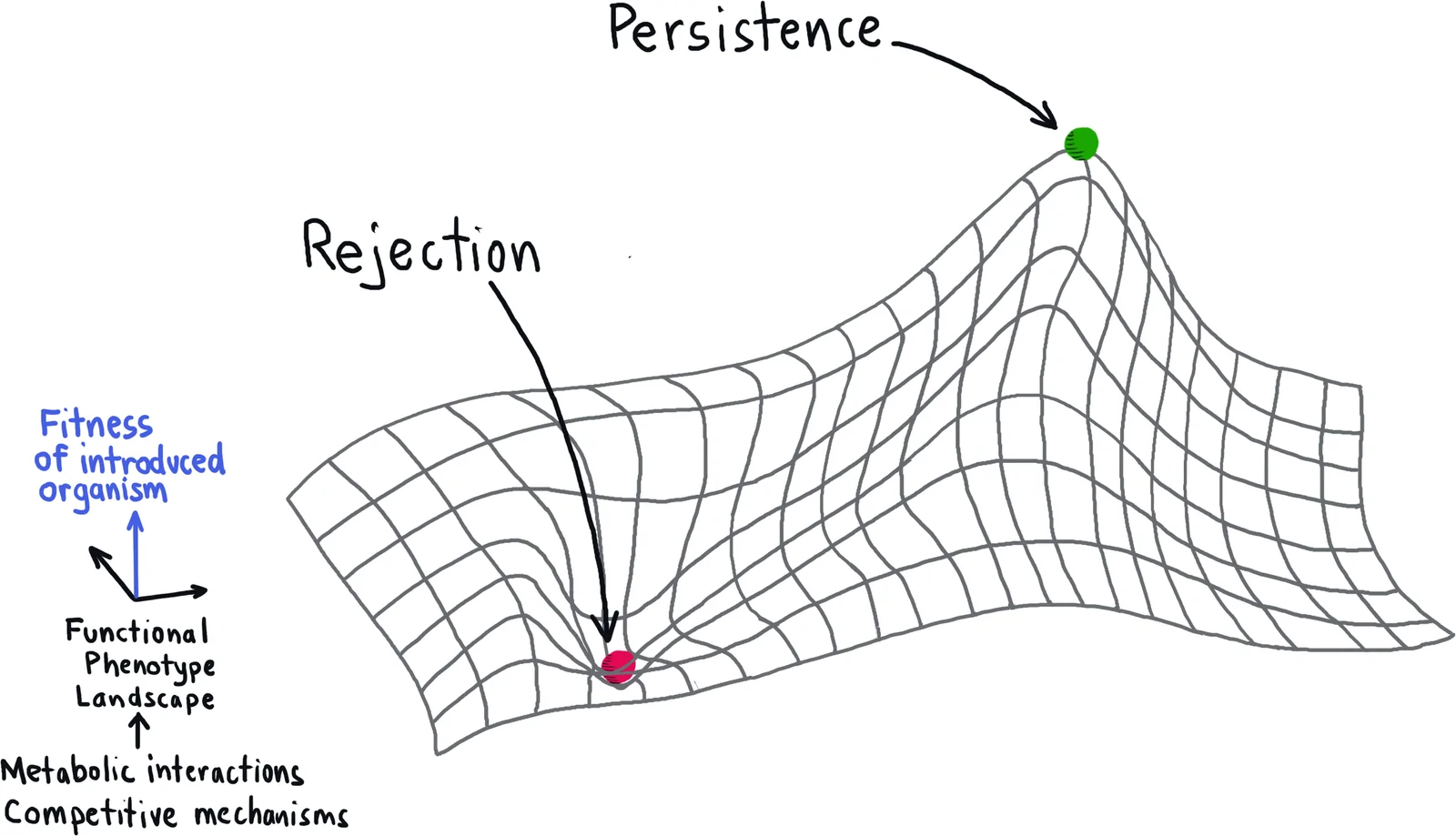
The concept of the persistence landscape describes how the combination of metabolic interactions (exchange and common goods) and competitive mechanisms leads to the fitness of an introduced organism, leading to either persistence (high fitness) or rejection (low fitness).

To understand and model the persistence of introduced organisms in a target community, we propose a framework to consider a persistence landscape (Fig. 1). Specifically, the persistence landscape focuses on consideration of two community properties that are important to the persistence of an introduced organism: competitive mechanisms and metabolic exchange and production of public goods. In this framework, a set of functional capabilities for organisms in the target community are defined, focusing on the competitive elements (antibiotic factors, etc.), the metabolic exchange potential, and the horizontal exchange propensity (conjugative transfer and phage). These functional persistence profiles are then used to calculate similarities between communities with different compositions to establish the landscape. The fitness of the specific engineered organism in the community with that composition defines the height of the landscape. This framing, combined with advances that have come from work on community function landscapes, provides a practical way of formulating this complex problem (21, 22) and presents a tractable approach that could be used to model the persistence landscape to enable identification and assessment of factors that support or hinder persistence while also informing containment strategies to restrict escape.

Realizing a persistence landscape model will require advances in computational annotation and modeling, which will provide a way to render the species composition of the target community into a functional profile that will best describe the landscape. As envisioned, a simple persistence landscape could be predicted from the genome sequences of the target community member species given existing metabolic and functional annotation tools. Improvement in community metabolic modeling approaches (24) will allow better identification of the metabolic exchange propensity of the target microbiome. Empirical methods, such as metabolomics, transcriptomics, and gene-tracking techniques, could be used to enhance this landscape to go beyond functional genome-based predictions (23, 25). Improved prediction of biological “dark matter” (26), specifically as it pertains to the identification of microbial defense and offense mechanisms (27), will improve the landscape. Finally, measurement of engineered organism persistence in many combinations of potential target communities would provide the landscape height measurements that could be used to refine the landscape. Application of emerging approaches to modeling global epistasis in community landscapes could significantly reduce the number of measurements needed to describe a persistence landscape, but these principles have not yet been demonstrated for the persistence of novel organisms in a community (21, 22). Integrating these approaches into a persistence landscape framework could offer microbiome researchers and ecologists a powerful tool to study and design microbiome engineering strategies.

## PRACTICAL ASSESSMENT OF PERSISTENCE LANDSCAPES

We provide two example use cases for determination of a persistence landscape in agronomy and biomanufacturing, but similar approaches could be envisioned in human microbiomes by using selective diet supplementation with rare carbon sources. First, a representative example of a target natural community for introduced organisms is the root rhizosphere, which is vitally important for plant growth (2, 28, 29). Understanding the persistence landscape of the rhizosphere, including the metabolic complementation potential of the host plant, will enable the design of engineered microbiomes with defined functional persistence traits. These traits include an increased ability to compete with native communities for rhizosphere resources and to minimize ecological impacts through containment in an agricultural context. Second is application to biomanufacturing, where designed communities offer significant advantages for increased yields and functional robustness in challenging environments (4, 7), but where rules for the introduction of new engineered species are not known.

Mapping the rhizosphere persistence landscape would inform strategies to increase colonization potential within the rhizosphere environment. One goal could be to identify metabolites that are not produced by other microbial constituents but which are *solely* produced by the plant, to provide mechanisms for biocontainment to the rhizosphere. The landscape would provide understanding of the metabolites produced by the community that could be targets for engineered pathways in the introduced organism. Similarly, prediction of how community-encoded defense mechanisms (antibiotic factors) might affect the persistence of the engineered organism would provide information for engineered countermeasures.

The rhizosphere comprises many thousands of species interacting in a complex spatial environment, making a comprehensive description of a persistence landscape experimentally intractable. We instead envision near-term experiments that focus on stable synthetic communities designed to represent key facets of the target community and an engineered organism. Following Sanchez-Gorostiaga et al. (23), the membership of the synthetic community would be varied and the persistence of an introduced organism measured. Enumerating community compositions (unique combinations of species) and their corresponding functional persistence profiles, as well as determining global epistasis, would allow definition of the persistence landscape, identification of combinations of species permissive or intolerant of an introduced organism, and sub-groups of community members that fulfill different functional roles and niche specialties.

Communities designed for biomanufacturing are already more tractable than natural communities, given the limited number of member species (5). For this application, determination of a persistence landscape would provide an actionable set of principles for the stability of engineered functions in the communities, by providing a recipe for enhancing persistence of the introduced organism by varying the composition of the community according to those principles. Such a persistence landscape could be coupled with a community function landscape by measuring a desired outcome, production of a precursor compound, for example, to allow maximization of persistence and thus functional stability, with the production of the compound. However, even in reduced complexity communities such as these, the combinatorial aspect could still be prohibitive to measure experimentally.

Advances in automated experimentation using robotic platforms offer an efficient way to approach the persistence landscape. Coupled with computational approaches to guide experimental design, automated platforms can be used to rapidly measure persistence phenotypes with large numbers of different community member combinations to define a persistence landscape for an engineered organism. The ability to derive functional effect equations and use them to extrapolate persistence impact will allow communities that are significantly more complex to be considered. While this has yet to be attempted with microbial communities, a similar framework employed an automated pipeline that used deep learning to predict combinations of amino acids that support the growth of a single organism (30). Mapping a persistence landscape presents additional challenges such as accounting for unculturable community members and a vastly larger combinatorial space, but similar approaches could enable its characterization for synthetic communities.

Safe and ethical deployment of engineered microorganisms in the environment requires consideration of the possibility that the organisms with engineered functions might survive and propagate outside the intended target community (1, 31). There are multiple known pathways for escape, including physical dispersal via wind, water, or animal vectors, as well as genetic dispersal through mechanisms such as horizontal gene transfer (1, 2, 32). Such dispersal can lead to unforeseen ecological consequences, including disruption of native microbial communities, alteration of key ecosystem functions, and the transfer of engineered traits to non-target species, creating new selective pressures or evolutionary trajectories (1). As a result, containment strategies are not only desirable but often essential to mitigate the risks of unintended consequences (33, 34).

We envision that the persistence landscape concept could be applied to non-target communities to determine the potential for support of the introduced organism that has escaped. A critical factor influencing efficacy in both colonization and containment is the ability of microbial communities to complement functions intentionally missing from an introduced organism. While functional complementation by target communities (e.g., rhizosphere bacteria providing essential nutrients to auxotrophic engineered strains) can enable successful colonization to supply beneficial functions such as plant growth promotion, complementation by non-target communities (e.g., bulk soil or waterways) could undermine containment strategies by allowing engineered organisms to proliferate beyond intended environments (1, 2). Determining a persistence landscape for other non-target communities, for example, soil surrounding a root rhizosphere community, provides a way of understanding and designing containment strategies for the engineered organism.

## CONCLUSION

The persistence landscape framework presented here provides a way of simplifying the complex set of factors that affect the persistence of organisms and their intended functions introduced in a target microbiome community. Multiple challenges must be addressed to develop and apply persistence landscapes in practice. The combinatorial explosion of species and functions is a significant challenge that can be addressed using careful application of synthetic communities and autonomous experimentation. Methods for data acquisition to understand the complex relationships between community members and between a community and an introduced organism are limited. This points to the need for higher-resolution measurements and high-fidelity genome-scale models to pinpoint how individual community members contribute to the landscape. Relatedly, computational models capable of representing the molecular interactions between the introduced organism, the target community members, and environmental factors of relevance are needed to realize the persistence landscape. Such models will need to be trained on large amounts of data from communities that could be greatly accelerated by AI-enabled automated experimentation systems, as described above. Addressing these challenges with a conceptual framework like the persistence landscape could usher in an era of engineered microbiomes with positive impacts on economies and ecosystems.
